# A comprehensive update on the cost-effectiveness of 10-year denosumab vs alendronate in postmenopausal women with osteoporosis in the United States

**DOI:** 10.1007/s11657-025-01564-x

**Published:** 2025-06-30

**Authors:** Eric Yeh, Matia Saeedian, Jack Badaracco

**Affiliations:** 1 Amgen Inc, Thousand Oaks, CA USA; 2 BluePath Solutions, Los Angeles, CA USA

**Keywords:** Alendronate, Cost-effectiveness, Denosumab, Fracture risk, Osteoporosis

## Abstract

***Summary*:**

In postmenopausal women with osteoporosis, 10-year denosumab was estimated to be cost-effective vs 5 years of oral alendronate, a 2-year drug holiday, and subsequently 3-years of alendronate with an estimated incremental cost-effectiveness ratio of $97,574 per quality-adjusted life-years gained. Cost-effectiveness was demonstrated in most of the scenario simulations.

**Purpose:**

A previous economic analysis estimated that 5-year denosumab was cost-effective compared with 5-year alendronate in women with postmenopausal osteoporosis (PMO) in the United States (US). Emerging literature has provided data on the long-term clinical benefits of denosumab. Therefore, the cost-effectiveness analysis was updated to understand the potential implications of a longer treatment duration (10-year) with denosumab vs generic oral alendronate or no treatment from a US third-party payer perspective.

**Methods:**

A lifetime Markov cohort model was used to compare 10-year denosumab treatment to 5 years of alendronate, followed by a 2-year drug holiday and, then an additional 3 years of alendronate. The target population consisted of PMO women in the US with a starting age of 72 years. Recent publicly available data, including epidemiology, treatment efficacy, persistence, and costs, were used to inform model inputs. Scenario analyses and a probabilistic sensitivity analysis (PSA) were conducted to account for uncertainty.

**Results:**

Estimated mean total lifetime cost and quality-adjusted life years (QALYs), respectively, were $81,003 and 8.035 for denosumab, and $75,358 and 7.977 for alendronate, resulting in denosumab having an incremental cost-effectiveness ratio of $97,574 per QALY gained. At a threshold of $150,000 per QALY, the PSA demonstrated that denosumab was considered cost effective in 62.1% of simulations. Denosumab was dominant over no treatment.

**Conclusions:**

Ten-year denosumab treatment would be cost-effective compared with 5 years of alendronate, followed by a 2-year drug holiday and 3 years of alendronate at the threshold of $150,000. Cost-effectiveness was demonstrated across most scenarios with robust PSA results.

**Supplementary Information:**

The online version contains supplementary material available at 10.1007/s11657-025-01564-x.

## Introduction

Osteoporosis is a chronic skeletal disorder characterized by compromised bone strength that predisposes an individual to an increased risk of fracture [1]. Approximately two-thirds of all osteoporosis-related fractures occur in women. It was estimated that the baseline 1-year incidence rate of any type of fragility fractures in patients aged ≥ 50 years without a history of prior fractures was 17.86 per 1000 women and 10.33 per 1000 men in the United States (US) [2]. These data estimated that a total of ≥ 1.72 million fragility fractures would occur in 2023 in the US general population aged ≥ 50 years, i.e., ≥ 197 new fractures per hour.

Osteoporotic fractures have physical, social, and psychological consequences that have a negative impact on health-related quality of life (HRQoL) and increase healthcare expenditures [3–5]. An international study (including US data) suggested that women aged ≥ 50 years with a recent fragility fracture (≤ 1 year) had significantly worse physical function, lower levels of productivity, and greater caregiver burden compared with women with no fractures [4]. A 68% increase (1.9 to 3.2 million) in annual fractures and ~ 67% increase ($57 to $95 billion) in overall related medical costs were projected from 2018 to 2040 in women with postmenopausal osteoporosis (PMO) aged ≥ 65 years in the US [6].

The American Association of Clinical Endocrinologists/American College of Endocrinology Clinical Practice (AACE/ACECP) 2020 guidelines recommend alendronate or denosumab as two options for the initial therapy for most osteoporotic patients with high fracture risk [7]. Denosumab is a fully human monoclonal antibody that specifically binds the receptor activator of nuclear factor-κB (RANK) ligand with high affinity and is a key mediator in reducing bone resorption [8]. In the US, denosumab is indicated for the treatment of PMO women at high risk of fracture. In the FREEDOM trial, denosumab reduced fracture risk by 68%, 20%, and 40% for vertebral, non-vertebral, and hip fractures, respectively, at 3 years [9, 10]. The 10-year FREEDOM extension study showed low fracture incidence and further increase in bone mineral density (BMD) with long-term denosumab at the lumbar spine, total hip, femoral neck, and one-third radius [11].

A previously published cost-effectiveness analysis reported the incremental cost-effectiveness ratio (ICER) of $85,100/quality-adjusted life-year (QALY) gained in the base case for 5-year treatment with denosumab compared with 5-year treatment of generic oral alendronate in PMO women in the US [12]. With data on the long-term clinical benefits of denosumab now available [11, 13, 14] and to understand the potential implications of an extended treatment duration, this study aimed to assess the cost-effectiveness of 10-year treatment with denosumab vs generic oral alendronate or no treatment in PMO women from a US third-party payer perspective.

## Methods

### Model overview

This study utilized a Markov cohort model adapted from the previously published denosumab cost-effectiveness model [12]. The target population was based on the FREEDOM trial, which consisted of PMO women [11]. In the base-case setting, key baseline characteristics included a starting age of 72 years, baseline *T*-score of − 2.5 standard deviations (SD), and prevalent vertebral fracture in 23.6% of patients. This study evaluated the costs and effectiveness of denosumab (subcutaneous injection of 60 mg every 6 months) vs “no treatment” and alendronate (oral 70 mg weekly) for a maximum of a 10-year window in the base-case analysis and varied on-treatment and drug holiday durations in different scenario analyses.

The model was conducted from a US third-party payer perspective over a lifetime horizon. The model’s primary outcome measure was ICER, represented as the incremental cost per QALY gained. The analysis also provided estimates for direct medical costs and life-years, and the cumulative incidence of hip, vertebral, wrist, and other fractures.

### Model structure

**Fig. 1:**
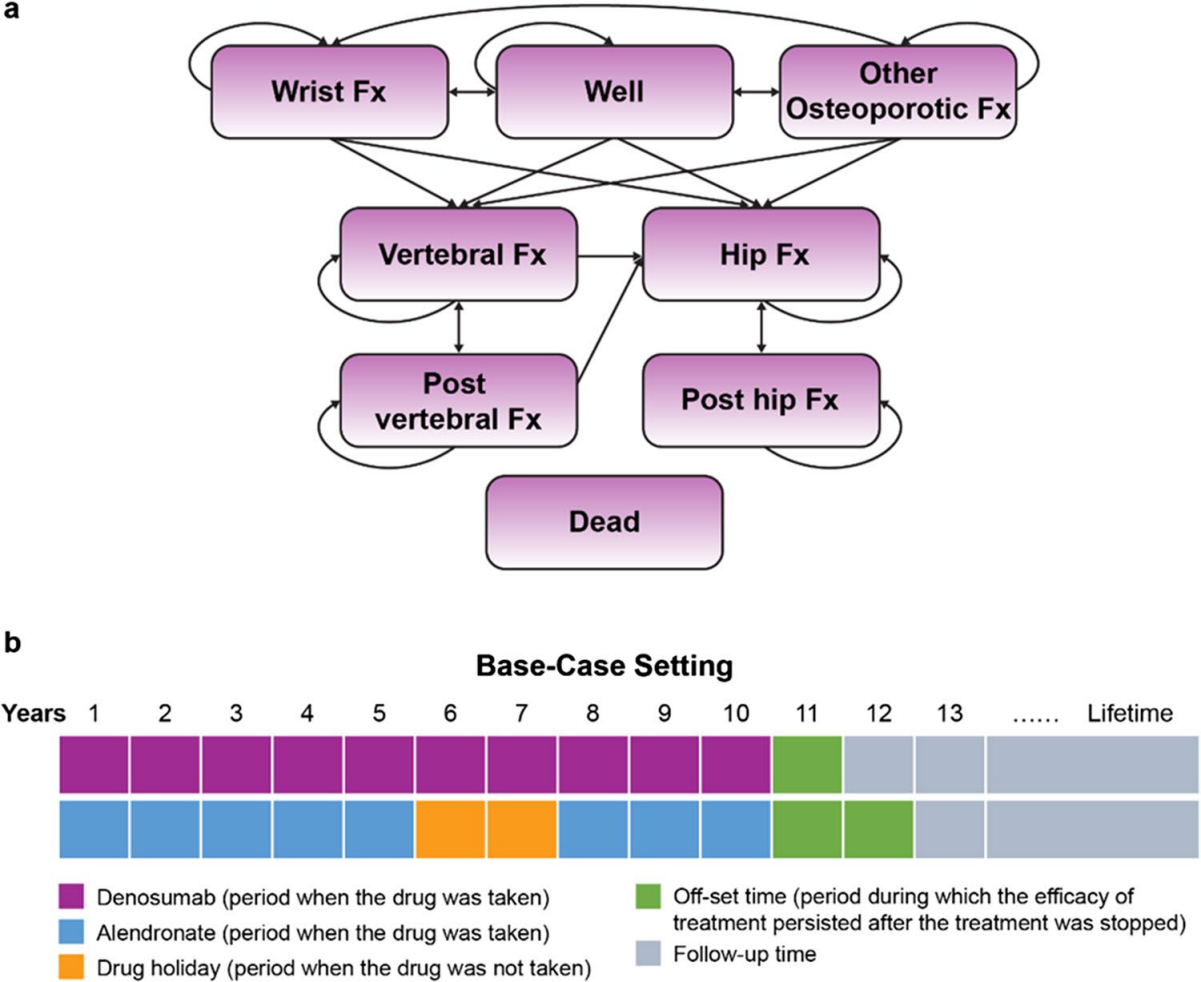
Model structure and study design. **a** Structure of the denosumab Markov cohort model. **b** Study design in the base-case setting. *Fx* fracture

The model consisted of eight health states: well, hip fracture, vertebral fracture, wrist fracture, other osteoporotic fracture, post vertebral fracture, post hip fracture, and dead (Fig. 1a). The model used 6-month cycles considering the following reasons: (1) Osteoporosis is a chronic condition; a longer cycle length is deemed appropriate for capturing outcomes; (2) denosumab is used every 6 months; and (3) fracture outcomes as a measure of treatment efficacy and real-world medication persistence data are reported at either a 6-month or 1-year interval.

The model used a hierarchical structure to appropriately capture HRQoL/utility measures associated with a particular fracture type, and this hierarchy was based on the severity of fracture types (considering the impact on HRQoL and costs), with hip fracture being the most severe, followed by vertebral fracture. Fracture outcomes were estimated per 6-month cycle; however, utility decrements associated with fractures in the study model were estimated over 1 year for a total of 2 cycles [5].

Depending on the fracture type, patients transitioned to the hip fracture, vertebral fracture, wrist fracture, or other osteoporotic fracture state and remained in the fracture health state. Patients in the wrist fracture or other fracture state who did not sustain another fracture return to the “well” state. Patients in the hip or vertebral fracture state may end up in one of the four scenarios at the next cycle, depending on the occurrence of a fracture and its type. For example, a patient in the hip fracture state may (a) transition to the “post hip fracture” state for recovery if no fracture occurs; (b) transition to the “post hip fracture” state with an adjustment for the downstream subsequent fracture if the patient experiences a subsequent vertebral, wrist, or other fracture; (c) stay in the “hip fracture” state if a subsequent hip fracture event occurs; or (d) transition to the “dead” state. In the scenario (a) or (b) where the patient transitions to the “post hip fracture” state, 1-year disutility of HRQoL and fracture costs apply to one cycle of the “hip fracture” state and one cycle of the “post hip fracture” state. Given the possibility that patients in the post hip or post vertebral state could still sustain other fractures, the model attempted to account for downstream fractures by multiplying the number of patients in each higher hierarchy state with the incidence rate of the lower hierarchy fracture type in the model population.

### Incidence of fractures

The fracture risk was assessed based on the method used previously [12] and is illustrated in Fig. S1. Fracture incidence depended on three factors: (i) the risk of incurring a fracture for an individual in the general population, (ii) the increased fracture risk associated with osteoporosis and a history of fractures (relative risk [RR]), and (iii) a risk reduction, if any, attributed to a treatment. The key model inputs used to estimate fracture risk are described in Table 1. The risk of experiencing a fracture in the model was calculated as follows:

**Table 1:** Key model inputs

Parameter		Values				Source
RR of fractures		Hip	Vertebral	Wrist	Others	
RR of fracture per SD of hip BMD		2.60	1.80	1.40	1.60	Marshall et al. (1996) [16]
RR of hip fractures per SD in men and women combined by age	65 years	2.89				Johnell et al. (2005) [17]
	70 years	2.78				
RR of fractures with a history of prior fracture Original		65 years 2.28	4.40	1.40	1.90	Kanis et al. (2004) [18] (for hip fracture) Klotzbuecher et al. (2000) [19] (for vertebral, wrist and other fractures)
		70 years 1.90				
Adjusted^a^			3.96	1.26	1.71	
Treatment efficacy (RR of fractures) Alendronate vs placebo	12 months	0.86	0.51	0.77	0.77	Willems et al. (2022) [13]
	24 months	0.51	0.37	0.53	0.53	
	36 months	0.52	0.51	0.62	0.62	
Denosumab vs placebo	12 months	0.55	0.39	0.84	0.84	Willems et al. (2022) [13]
	24 months	0.47	0.29	0.79	0.79	
	36 months	0.28	0.33	0.82	0.82	
Treatment persistence						
Alendronate	0	100.0%				Singer et al. (2021) [20]
	at 6 months	56.1%				
	at 12 months	38.8%				
	at 18 months	29.8%				
	at 24 months	24.7%				
	at 30 months	20.3%				
	at 36 months	17.2%				
Denosumab	0	100.0%				Singer et al. (2021) [20]
	at 6 months	93.6%				
	at 12 months	72.7%				
	at 18 months	59.6%				
	at 24 months	50.3%				
	at 30 months	43.2%				
	at 36 months	38.0%				
Resource use, unit costs, and utilities						
Treatment monitoring/administration costs^b^	BMD measurement: CPT code 77,080	$38.95				2024 CMS [21]
	Physician visit: CPT code 99,213	$90.87				
	IV injection: CPT code 96,365	$62.58				
	Nurse visit: CPT code 96,372	$14.31				
Annual medication costs	Alendronate	$44				NAVLIN Pricing and Reimbursement data [22]
	Denosumab	$2,899				
Costs of fractures^a^ (in first and subsequent years)	Hip—year 1	$67,115 (age 50–64 years) $51,760 (age ≥ 65 years)				Tran et al. (2021) [23], inflated to 2024 USD
	Hip—year 2 +	$11,145 (age 50–64 years) $7,896 (age ≥ 65 years)				
	Vertebral—year 1	$33,923 (age 50–64 years) $23,949 (age ≥ 65 years)				
	Vertebral—year 2 +	$8,455 (age 50–64 years) $5,943 (age ≥ 65 years)				
	Wrist or Other—year 1	$16,279 (age 50–64 years) $21,731 (age ≥ 65 years)				
Long-term care costs (per day)	Nursing home	$216				Teigland et al. (2023) [24], inflated to 2024 USD
Age-specific health state utilities (women)	50–59 years	0.837				Hanmer et al. (2006) [25]
	60–69 years	0.811				
	70–79 years	0.771				
	80 + years	0.724				
Quality of life multiplier (in first and subsequent years)	Hip, year 1	0.550				Svedbom et al. (2018) [5]
	Hip, year 2 +	0.860				
	Vertebral year 1	0.680				
	Vertebral, year 2 +	0.850				
	Wrist or other, year 1	0.830				

^a^RR values were adjusted downwards by 10% to adjust for BMD

^b^Cost adjusted to 2024 USD

*BMD* bone mineral density, *CMS* centers for medicare and medicaid services, *CPT* current procedural terminology, *IV* intravenous, *RR* relative risk, *SD* standard deviation, *USD* US Dollars

$$\left(\text{risk in the general population}\right) \times \left(\text{elevated RR of fracture due to low BMD or prior fractures}\right) \times (\text{risk reduction from treatment})$$

In this updated version of the model, the long-term consequences of prior vertebral fractures on the acute hip fracture and post hip fracture states were estimated. The estimation was performed by approximating the proportion of patients experiencing the consequences of a hip fracture who also sustained a vertebral fracture within the relevant time period (lifetime for health utility, and 8 years for mortality). First, the incremental proportion of patients with vertebral fracture was divided by the proportion of patients without a prior history of vertebral fracture at the beginning of the considered period. Subsequently, using this proportion for weighting, the RR for increased mortality was applied post fracture.

### Treatment efficacy

In the base-case, treatment efficacy data, that is, pooled RR of fractures at different skeletal sites at 12, 24, or 36 months, were used from a network meta-analysis (NMA) [13] (Table 1) to inform fracture occurrences for alendronate or denosumab vs placebo. For the fourth year and beyond, RRs were assumed to remain consistent with the 36-month data. Variability in the fracture reduction benefit of treatment could impact model outcomes; to explore the impact of such uncertainties, the model also included data from two alternative NMA in the sensitivity analysis [14, 15] (Table S1).

### Persistence

In the base-case, key persistence data were derived from Singer et al. (2021) study [20] (Table 1). Persistence was modelled in each 6-month cycle. The proportion of patients persistent with denosumab was updated every 6 months in alignment with denosumab administration, while persistence in the alendronate arm was estimated from the midpoint of the cycle. Persistence rates were incorporated to estimate the benefit of fracture reduction and cost of treatment. In patients who were not persistent, fracture rates were assumed to return to the same level as untreated patients following an “offset time.”

As the study duration was 10 years and no study provided long-term persistence estimates in real-world settings, persistence was extrapolated by assuming a discontinuation rate equivalent to the last observed value in the base-case. Alternative options such as last interval rate (last available interval of data) and overall rate (start to the end of the data to calculate a rate that is interpolated) were also used for the sensitivity analysis.

### Treatment offset time

After treatment was discontinued, treatment efficacy was assumed to decrease continuously within a period of time (i.e., offset time) in a linear relationship until the fracture rates return to the baseline value. When patients discontinued the treatment early, they may have experienced partial health benefits and a shorter treatment efficacy offset time, which could affect the number of sustained fractures and mortality, and consequently the costs and HRQoL. In our model, the offset time for alendronate was set to 2 years, as a drug holiday of > 2 years is associated with an increased risk of hip, humerus, and clinical vertebral fractures [26]. For denosumab, offset time was established as 1 year in the base-case scenario, which was informed by insights gathered from a comprehensive narrative review [27]. This highlighted that upon denosumab discontinuation, patients experienced a rapid reversal of bone-protective effects of denosumab, leading to significant bone turnover and loss, and an increased risk of fractures, especially vertebral fractures. Alternatively, in the sensitivity analysis, the offset period for denosumab was adjusted to 0 or 2 years to account for the bone turnover and loss after denosumab discontinuation, and alendronate for 1, 3, or 5 years.

### Mortality

Age-specific all-cause mortality for women was taken from the period life table for the 2021 US population, as reported in the 2024 trustees report [28] (Table S2). To account for excess mortality due to fractures, the age-specific RR of mortality was applied to general mortality rates for hip, vertebral, and other fractures [29] (Table S3). In line with previous economic analyses of osteoporosis treatments, it was assumed that 30% of the excess mortality following a fracture was attributable to the fracture itself and the increased risk of mortality following hip and vertebral fractures persisted for 8 years after the event [12, 29–31]. In addition, alternative values (min 0%, 18%, 42%, and max 100%) for excess mortality related to fracture were used for sensitivity analyses [32, 33].

### Resource use and costs

The model included the cost of drug intervention and administration, cost of treating fractures, monitoring costs, and long-term care costs as shown in Table 1 [5, 20–25]. Medical costs of treating fractures included costs in the first year and the subsequent years following hip, vertebral, and other fractures. Costs of wrist fractures were assumed to be equivalent to those of other or non-vertebral fractures, including rib, clavicle, scapula, and sternum fractures. Acute (1 year) costs incurred due to additional downstream fractures were estimated by multiplying the number of additional fractures in each cycle with the 1-year costs of the fracture type (Table 1). Long-term care costs applied only to patients entering a nursing home following a hip fracture, but not wrist, vertebral, and other fractures [34, 35]. All costs were inflated to 2024 USD using the medical care consumer price indices [36].

### Health-related quality of life

Utility scores for women from the general population in the US [25] were used to inform age-specific health state utilities for patients in the “baseline” state (Table 1). To account for the HRQoL loss due to fracture in the first year after hip, vertebral, and other fractures, and in the second and subsequent years after hip and vertebral fracture, utility multipliers were applied to general population utilities [5]. Model inputs were informed with utility scores, before and after a fracture, for different fracture locations and at different time points.

The acute disutilities (i.e., an absolute health utility decrement) of the downstream fractures were accounted for in three steps. First, the HRQoL for a lower hierarchy fracture was derived by multiplying the HRQoL of the higher hierarchy fracture with the utility multiplier of the lower hierarchy fracture. Thereafter, a disutility of the downstream fracture was estimated by calculating the differences between the two HRQoL scores. Finally, the QALYs accrued in each cycle were adjusted downward using the estimated disutility.

### Analyses

#### Base-case and scenario analysis

The analysis estimated the total discounted lifetime costs and QALYs for each intervention, and the incremental cost (∆cost) per additional QALY gained (∆QALY) to yield the ICER (calculated as ∆cost/∆QALY), for denosumab vs no treatment and denosumab vs alendronate (Table 2). Both regimens had a maximum treatment duration of 10 years with the alendronate regimen including a drug holiday of 2 years after 5 years on-treatment, followed by a 3-year on-treatment period (Fig. 1b).

**Table 2:** Base-case cost-effectiveness analysis of a 10-year treatment with denosumab vs no treatment or alendronate

	Denosumab	No treatment	Alendronate	Difference Denosumab vs no treatment	Difference Denosumab vs alendronate
Total cost (US$)	81,003	81,676	75,358	− 674	5645
Cost of hip fractures	40,043	47,018	43,493	** − **6976	**–**3450
Cost of vertebral fractures	16,915	20,591	18,472	** − **3676	**–**1557
Cost of wrist fractures	6184	6444	6012	** − **260	172
Cost of other fractures	7312	7623	7108	** − **311	204
Drug cost	10,066	0	78	10,066	9988
Treatment management cost	483	0	195	483	287
Fracture events					
Hip fractures	0.3562	0.3981	0.3757	** − **0.0419	− 0.0195
Vertebral fractures	0.3993	0.4614	0.4232	** − **0.0621	− 0.0239
Wrist fractures	0.2784	0.2790	0.2628	** − **0.0006	0.0156
Other fractures	0.3300	0.3310	0.3115	** − **0.0010	0.0184
Any fractures	1.3638	1.4695	1.3732	** − **0.1057	− 0.0094
QALYs and life years					
Life-years (discounted)	11.352	11.296	11.352	0.056	0.026
QALYs	8.035	7.903	7.977	0.133	0.058
∆Cost per ∆QALY gained (US$)				Denosumab is dominant	97,574

*QALY* quality-adjusted life year

**Fig. 2:**
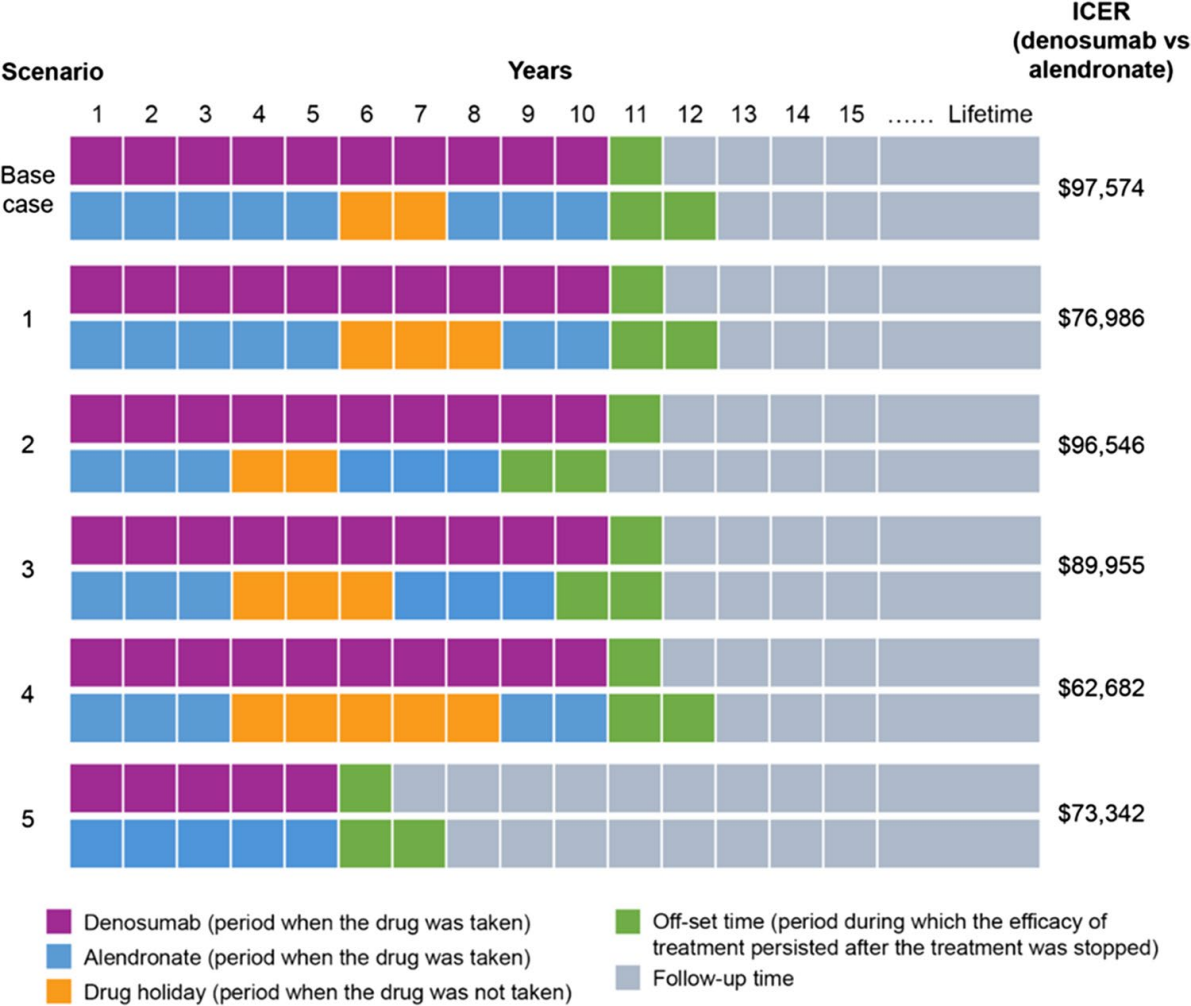
Cost-effectiveness results: scenario analysis. Cost-effectiveness analyses of different scenarios with varying on-treatment and drug holiday durations and their respective ICERs. *ICER* incremental cost-effectiveness ratio

For scenario analyses, various combinations of on-alendronate treatment and drug holiday durations were tested, keeping the denosumab regimen constant, and ICERs were estimated for these scenarios (Fig. 2).

#### Sensitivity analysis

To account for the uncertainty and to test the robustness of the analysis, we conducted a one-way sensitivity analysis by varying the value of each key variable at a time (Table 3), and a probabilistic sensitivity analysis (PSA) by varying all variables together. In the one-way sensitivity analysis, some of the varied inputs included starting age, baseline BMD, treatment efficacy, treatment duration, denosumab offset time, persistence, and discount rates (Table 3).

**Table 3:** One-way sensitivity analysis for cost-effectiveness of treatment with denosumab vs no treatment and denosumab vs alendronate

Variable	Base-case value	Alternative value	ICER vs no treatment	ICER vs alendronate
Base-case setting			Cost saving	$97,574
Start age	72 years old	50 years old	$34,957	$175,341
		65 years old	$6,992	$98,160
		80 years old	Cost saving	$54,482
Prevalent fracture	23.60%	0%	$13,281	$154,596
		100%	Cost saving	$34,515
Baseline BMD *T*-score	≤ − 2.5	≤ ** − **2.8	Cost saving	$74,224
		≤ ** − **3.0	Cost saving	$59,678
Pooled treatment efficacy	Willems et al. (2022) [13]	Freemantle et al. (2013) [40]	$18,712	$131,309
		Barrionuevo et al. (2019) [15]	$12,075	$114,621
		Ayers et al. (2023) [14]	$17,614	$190,093
Modeling horizon	Lifetime	10 years	$47,395	$219,335
		15 years	$5,423	$125,949
		20 years	Cost saving	$103,476
Maximal treatment length	10 years	5 years	$2504	$73,342
		Lifetime	Cost saving	$91,522
Denosumab offset time	1 year	0 years	$2510	$142,579
		2 years	Cost saving	$77,744
Alendronate offset time	2 years	1 year	Cost saving	$77,385
		3 years	Cost saving	$104,376
		5 years	Cost saving	$113,852
Persistence extrapolation (long term)	Last value	5-year rate	Cost saving	$137,570
		Last interval rate	Cost saving	$97,574
		Overall rate	Cost saving	$166,698
Persistence	At 36 months: Denosumab, 38% Singer et al. (2021) [20], Alendronate, 17.2% Singer et al. (2021) [20]	At 36 months: Denosumab, 50.7% Borek et al. (2019) [41] Alendronate, 24.6% Li et al. (2012) [42]	Cost saving	$66,553
		At 24 months: Denosumab, 41.2% Durden et al. (2017) [43], Alendronate, 23.7% Durden et al. (2017) [43]	Cost saving	$110,405
		Assumption: Denosumab and Alendronate: 100%	Cost saving	$255,854
Discounting rate	3%	0%	Cost saving	$70,949
		5%	$2858	$116,125
Excess mortality related to fracture	30%	0%	Cost saving	$121,248
		18%	Cost saving	$105,180
		42%	Cost saving	$91,558
		100%	$8862	$74,347
Years of post hip/vert mortality	8 years	3 years	Cost saving	$101,780
		5 years	Cost saving	$99,006
		10 years	Cost saving	$97,310
		Lifetime	Cost saving	$97,205
Key inputs from Parthan et al. (2013) [12]	Base case value		$25,424	$78,591

Additional inputs: Number of nurse visits/year for denosumab, 2; number of physician visits/year, all treatments, 1; number of DXA scans/year, all treatments, 0.5; maximum offset time for alendronate, 2 years; efficacy offset assumption, dynamic; years of increased post-fracture mortality, 8 years. *BMD* bone mineral density, *DXA* dual-energy x-ray absorptiometry, *ICER* incremental cost effectiveness ratio

In the PSA, input parameters were stochastically varied simultaneously according to probability distributions representing their uncertainty [37]. Probability distributions were informed by parameter point estimates, standard errors (taken from sources if available, otherwise assumed to be 10% of point estimates), and nature of the parameter. Treatment efficacy estimates (RRs of fracture) were assigned a log normal distribution since ratios were asymmetrically distributed. Treatment persistence estimates, proportion of patients entering long-term care after hip fracture, and utilities were assigned a beta distribution, since these values typically lie between 0 and 1. Fracture costs were assigned a normal distribution, since these inputs could theoretically take any value (as fracture costs comprise the incremental healthcare expenditure for individuals who have sustained a fracture, vs those without a fracture).

The PSA sampled 1000 probabilistic iterations and obtained a distribution of model outcomes, which provided a probability that an intervention is cost-effective at a particular willingness-to-pay threshold [38, 39]. Incremental costs (∆cost) and incremental QALY (∆QALY) from the simulations were plotted and illustrated as cost-effectiveness acceptability curves (CEACs), where the proportion of iterations in which the cost-effectiveness of denosumab vs no treatment or denosumab vs alendronate was plotted across a range of cost per QALY thresholds.

## Results

### Base-case

Base-case results for the comparison of denosumab vs no treatment showed that the average incidence of any fracture with denosumab was expected to be lower by 0.1057 per patient vs no treatment (Table 2). This fracture reduction translated into a lifetime gain of 0.056 discounted life years and 0.133 discounted QALYs per patient. Compared with no treatment, denosumab was associated with a higher drug cost but lower total cost ($674 lower per patient) and was dominant over no treatment (Table 2).

In this deterministic analysis, results for the comparison of denosumab vs alendronate showed that the average incidence of any fracture with denosumab was expected to be lower by 0.0094 per patient compared with alendronate (Table 2). This fracture reduction translated into a lifetime gain of 0.026 discounted life years and 0.058 discounted QALYs per patient. Compared with patients on alendronate, those on denosumab incurred an additional cost of $5645, primarily due to a higher drug acquisition cost and treatment management cost. Denosumab therefore produced an ICER of $97,574 per QALY gained vs alendronate (Table 2).

### Scenario analysis

ICERs (denosumab vs alendronate) in scenarios of various durations of treatment and/or drug holiday are shown in Fig. 2. A shorter treatment duration of alendronate before the drug holiday or an increased drug holiday duration for alendronate led to a lower ICER compared with the ICER in the base-case. Using the current model, a comparison of 5-year denosumab vs 5-year alendronate (Fig. 2), similar to the one drawn in Parthan et al. (2013), demonstrated that the ICER ($73,342 = ∆cost of $3,343/∆QALY of 0.0461) reported in this study was lower than the ICER ($85,060 = ∆cost of $2,984/∆QALY of 0.0351) reported previously [12].

Additional inputs: Number of nurse visits/year for denosumab, 2; number of physician visits/year, all treatments, 1; number of DXA scans/year, all treatments, 0.5; maximum offset time for alendronate, 2 years; efficacy offset assumption, dynamic; years of increased post-fracture mortality, 8 years.

### Sensitivity analysis

One-way sensitivity analyses demonstrated that ICERs (denosumab vs alendronate) were most sensitive to assumptions regarding treatment persistence, efficacy data, drug holiday duration, and treatment offset time (Table 3). Reducing the denosumab offset time to 0 years reduced the residual effect of denosumab, thereby increasing the ICER. Reducing the on-treatment duration of alendronate increased the ICER, while increasing the drug holiday duration reduced the ICER. Reducing the offset time of alendronate to 1 year reduced the ICER, while increasing the offset time to 3 or 5 years increased the ICER.

**Fig. 3:**
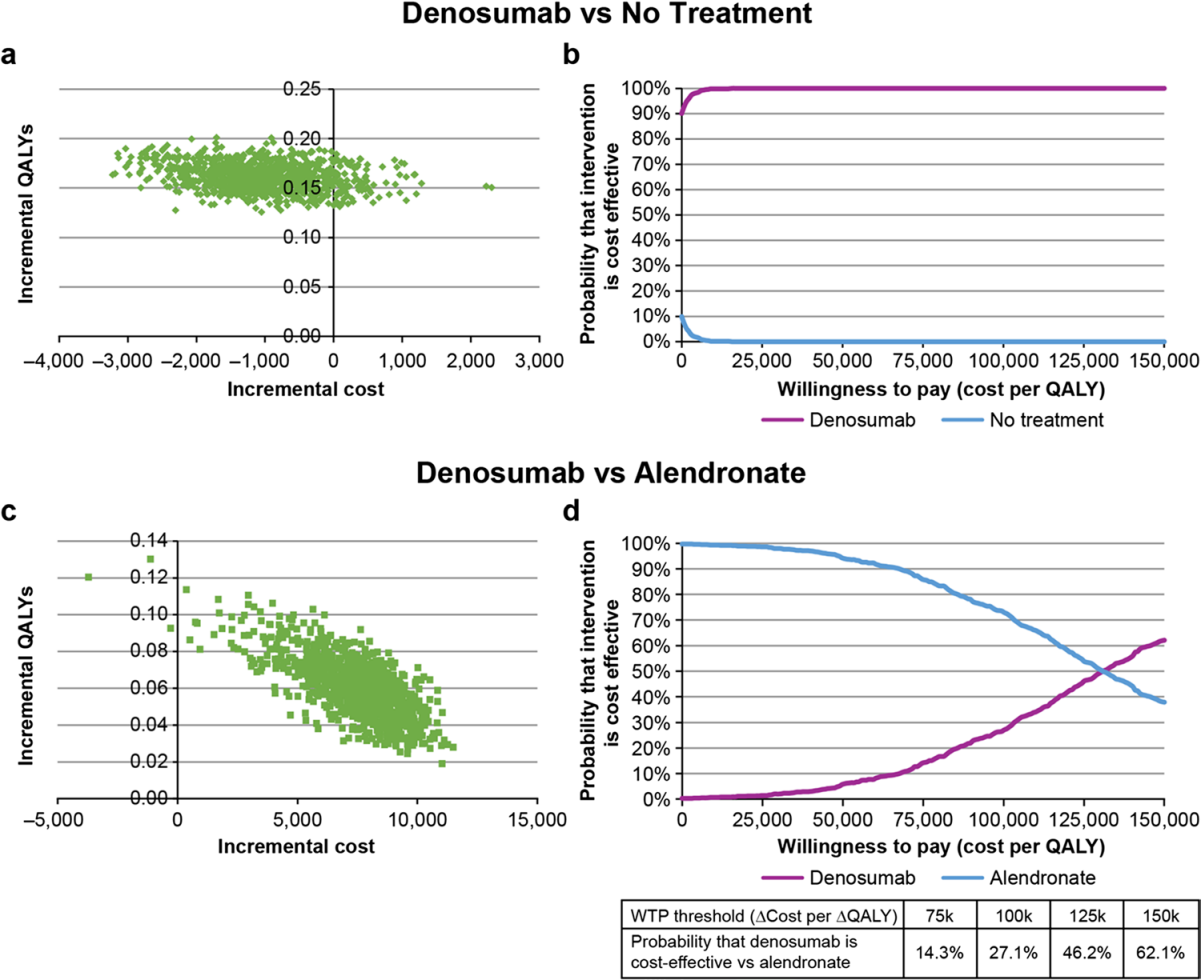
Probabilistic sensitivity analysis for denosumab vs no treatment and denosumab vs alendronate. **a** Scatterplot of incremental QALYs vs incremental cost for 1000 probabilistic samples for denosumab vs no treatment. **b** Cost-effectiveness acceptability curves for denosumab and no treatment. **c** Scatterplot and **d** cost-effectiveness acceptability curves for denosumab vs alendronate. *QALY* quality adjusted life year

PSA demonstrated that denosumab was cost-effective in 100% of probabilistic iterations at or above a cost per QALY threshold of ~ $10,000 per QALY gained compared with no treatment (Fig. 3). At a willingness-to-pay threshold of $100,000 and $150,000 per QALY gained, PSA demonstrated that denosumab had a probability of 46.2% and 62.1%, respectively of being cost-effective compared with alendronate (Fig. 3).

## Discussion

In this study, the economic evaluation of long-term denosumab treatment (10-year) was found to be dominant over no treatment and cost-effective compared with generic oral alendronate in PMO women in the US. The 1-year cost of denosumab was higher than that of alendronate, but the cumulative costs of denosumab would be partially offset by potential cost savings due to avoided incident fractures over time. Sensitivity analyses suggested that ICERs were sensitive to treatment-related parameters, such as treatment efficacy, offset time, and treatment persistence. However, PSA indicated that 10-year denosumab treatment was cost-effective in almost half the simulations at a willingness-to-pay threshold of $100,000 and in most cases at a threshold of $150,000 per QALY gained. Thus, these findings are important from a payers’ as well as clinicians’ perspective, i.e., a 10-year treatment with denosumab compared with alendronate would lead to lower fracture incidences, increased QALYs, and cost-effectiveness in PMO women. In addition, this long-term economic evaluation provides insights to policy makers to support informed resource allocation decision-making.

Our findings demonstrate that 10-year denosumab produced cost-effective ICERs (ranging from $62,682 to $97,574per QALY gained; Fig. 2) in different scenarios in comparison with generic oral alendronate. Compared to the previous version [12], the updated model used time-dependent treatment efficacy data from recent NMAs and recent real-world persistence data, which could be extrapolated over long-term, considered bone turnover and loss after denosumab discontinuation (adjusted via denosumab offset time), incorporated drug holiday for bisphosphonates, accounted for downstream fractures, and allowed various scenarios such as variations in treatment duration, drug holiday, and treatment offset time, which was unique. Using the same inputs and 5-year treatment duration as in the Parthan et al. (2013) study, our updated model produced an ICER of $88,720 per QALY gained, which is slightly different from the previously reported ICER of $85,060 [12].

Different treatment options have different efficacy in terms of reducing the fracture risk, which may vary at specific time points, in turn affecting the economic evaluation of treatment options [13]. In this study, we found that using RRs (12, 24, and 36 months) from a time-point specific NMA for base-case setting provided a lower ICER compared with those from other NMAs where RRs were not time-dependent [13–15, 40]. One of these NMAs combined oral alendronate with intravenous bisphosphonates (such as zoledronic acid) while estimating the fracture risk, which may have overestimated the efficacy of oral alendronate such that the fracture risk estimates over time may be less accurate [14]. Thus, the studies used to inform the RR in the sensitivity analysis may have overestimated the treatment efficacy in the alendronate arm, resulting in small differences between the two treatment arms and thus estimating a higher ICER in the sensitivity analysis compared with that in the base-case analysis.

Another critical factor influencing cost-effectiveness outcomes is treatment persistence. Real-world treatment effectiveness may be different from the efficacy reported in RCTs. Our study model considered the real-world persistence data [20, 41–43] and thus provided realistic estimates of benefit of fracture reduction and cost-effectiveness. Our findings demonstrated that denosumab, compared with alendronate, remained cost-effective with various persistence inputs [20, 41–43], except for an unrealistic case when the patient population was 100% persistent for the whole treatment period. Persistence rates decreased more rapidly with alendronate vs denosumab. Sensitivity analyses using alternate methods to extrapolate long-term persistence indicated that using an average 5-year or overall rate for extrapolation would over-estimate the persistence for alendronate over the long term, thus overestimating the anti-fracture efficacy in the alendronate arm. As a result, this diminishes the disparity in fracture outcomes between the two treatments, leading to a higher ICER estimate for denosumab.

From a clinical standpoint, discontinuation of denosumab results in rapid bone resorption, and early discontinuation can lead to a shorter treatment offset time and therefore lower treatment efficacy. Reducing the offset time from 1 year to zero in the sensitivity analysis decreased the overall efficacy of denosumab, resulting in a higher ICER, whereas extending the offset time to 2 years prolonged the residual effects of denosumab and thus widened the differences in treatment benefit between the two comparators leading to lower ICERs. Similarly, using longer offset time for alendronate (> 2 years) prolonged the residual effect of alendronate and thus reduced the differences in treatment benefit between the two comparators leading to higher ICERs.

The findings from this study should be interpreted considering the assumptions made in terms of the model structure and model inputs. First, the hierarchical nature of the Markov cohort model imposed some structural limitations, which led to an underestimation of the number of vertebral, wrist, and other fractures. Therefore, an adjustment was introduced to correct the omitted lower hierarchy fractures. However, even with this adjustment, it is possible that our model may still underestimate fracture outcomes, but the missing fractures should be minimal. This approach in fact is conservative and not favorable towards denosumab. In addition, although the model accounted for the occurrence of a prior fracture while estimating RR, the time since prior fracture (such as imminent fracture risk with a history of fractures in the past 2 years vs beyond 2 years) was not incorporated. Although the model used RR of fractures by treatment length at a 1-year interval (e.g., 12, 24, 36 months and beyond), it did not compute RR differently for patients discontinuing treatment at 6 months, rather used the same at 12 months. This could have overestimated fracture risk reduction in patients who discontinued treatment at 6, 18, or 30 months. Since the persistence rate was lower in the alendronate arm as compared with those in the denosumab arm, overestimation of fracture reduction would be higher in the alendronate arm and the ICER results would be conservative, and more in favor of alendronate and less favor of denosumab.

Second, there was uncertainty in the duration of treatment offset time, which impacted the treatment efficacy of denosumab, thereby impacting the ICERs. Third, while inputs were taken from US sources where available, data from international sources were used when appropriate US-specific data were not available, particularly to inform the HRQoL loss due to fracture, treatment persistence in the sensitivity analysis, and increased fracture risk in target patient populations with osteoporosis and/or prior fracture [5, 18, 42]. Fourth, persistence interpolation was based on persistence rates available for the first 3 years of treatment. This may have led to overestimating treatment persistence beyond 3 years in the model. Finally, the model assumed that patients receiving alendronate would take a 2-year drug holiday after 5 years. However, the recommended duration of drug holidays may vary in a real-world setting; a longer drug holiday would decrease the overall efficacy of alendronate, and a shorter holiday would increase efficacy and eventually would affect the ICERs.

## Conclusions

The results from this study suggest that 10-year long-term treatment with denosumab would be dominant over no treatment and cost-effective compared with generic oral alendronate in postmenopausal women with osteoporosis in the US with characteristics similar those in the FREEDOM trial. Higher persistence and lower fracture incidence rates would result in a gain in QALYs and a relatively low ICER compared with no treatment or alendronate. Finally, this study suggests that from a US third-party payer perspective, prolonged treatment with denosumab may effectively lower the economic burden of osteoporosis.

## Supplementary Information

Below is the link to the electronic supplementary material.

Supplementary file1 (DOCX 159 KB)
