# Supplementary material for: A comprehensive update on the cost-effectiveness of 10-year denosumab vs alendronate in postmenopausal women with osteoporosis in the United States
Source: Arch Osteoporos. 2025 Jun 30;20(1):85. doi: 10.1007/s11657-025-01564-x (PMC12209382; doi:10.1007/s11657-025-01564-x)
Supplement: Supplementary file 1 — Supplementary file1 (DOCX 159 KB) [file 11657_2025_1564_MOESM1_ESM.docx]

**SUPPLEMENTARY APPENDIX**

**Title:** **A Comprehensive** **Update on the Cost-Effectiveness of 10-Year Denosumab vs Alendronate in Postmenopausal Women With Osteoporosis in the United States**

**Journal:** *Archives of Osteoporosis*

**Authors:** Eric Yeh, Matia Saeedian, Jack Badaracco

**Corresponding author**

Eric Yeh, Amgen Inc., Thousand Oaks, California, USA

Email: [eyeh01@amgen.com](mailto:eyeh01@amgen.com)

**Table S1.** Fracture rates and relative risk of fractures

| **Parameter** | **Subgroup** | **Value** | | | | **Source** |
| --- | --- | --- | --- | --- | --- | --- |
|  |  | **Hip** | **Vertebral** | **Wrist** | **Others** |  |
| Annual incidence rates in the US female general population (per person year)^a^ | 50–54 | 0.000392 | 0.001961 | 0.004493 | 0.006393 | Yeh et al (2025) [2] |
|  | 55–59 | 0.000936 | 0.003366 | 0.006721 | 0.010091 |  |
|  | 60–64 | 0.001973 | 0.005613 | 0.009191 | 0.015074 |  |
|  | 65–69 | 0.002523 | 0.006260 | 0.010005 | 0.016763 |  |
|  | 70–74 | 0.003975 | 0.008387 | 0.011203 | 0.020528 |  |
|  | 75–79 | 0.006869 | 0.012377 | 0.014252 | 0.028482 |  |
|  | 80–84 | 0.012131 | 0.018722 | 0.019411 | 0.041201 |  |
|  | 85+ | 0.024113 | 0.028274 | 0.030053 | 0.065389 |  |
| Annual prevalence of vertebral fractures in the US | 50–59 |  | 0.033 |  |  |  |
|  | 60–69 |  | 0.046 |  |  |  |
|  | 70–79 |  | 0.106 |  |  |  |
|  | 80+ |  | 0.163 |  |  |  |
| Treatment efficacy: RR (95% Cl) of fractures (alendronate vs placebo) | 1–36 months | 0.65 (0.43**–**0.97) | 0.46 (0.24**–**0.89) | 0.68 (0.51**–**0.92) | 0.68 (0.51**–**0.92) | Ayers et al (2023) [14] |
|  | ≥ 36 months | 0.64 (0.50**–**0.82) | 0.38 (0.24**–**0.62) | 0.79 (0.68**–**0.91) | 0.79 (0.68**–**0.91) |  |
| Treatment efficacy: RR (95% Cl) of fractures (denosumab vs placebo) | 1–36 months | 0.61 (0.37**–**0.98) | 0.32 (0.21**–**0.48) | 0.81 (0.69**–**0.96) | 0.81 (0.69**–**0.96) |  |
|  | ≥ 36 months | 0.61 (0.37**–**0.98) | 0.32 (0.21**–**0.48) | 0.81 (0.69**–**0.96) | 0.81 (0.69**–**0.96) |  |
| RR (95% Cl) of fractures (alendronate vs placebo) |  | 0.61 (0.42**–**0.90) | 0.57 (0.45**–**0.71) | 0.84 (0.74**–**0.94) | 0.84 (0.74**–**0.94) | Barrionuevou et al (2019) [15] |
| RR (95% Cl) of fractures (denosumab vs placebo) |  | 0.56 (0.35**–**0.90) | 0.32 (0.22**–**0.45) | 0.80 (0.67**–**0.96) | 0.80 (0.67**–**0.96) |  |

^a^Data were based on Optum CDM claims databases from January 2023 to December 2023. Values were linearly interpolated or extrapolated as required to provide rates for each year of age, assuming that reported values correspond to the age at the midpoint of each group.

CDM, Clinformatics Data Mart; RR, relative risk.

Table S2. US mortality rates in the general population by age^a^

| **Age (Years)** | **Death Rate** | **Age**  **(Years)** | **Death Rate** | **Age**  **(Years)** | **Death Rate** | **Age**  **(Years)** | **Death Rate** | **Age**  **(Years)** | **Death Rate** |
| --- | --- | --- | --- | --- | --- | --- | --- | --- | --- |
| 50 | 0.004011 |  |  |  |  |  |  |  |  |
| 51 | 0.004306 | 61 | 0.009411 | 71 | 0.018859 | 81 | 0.051334 | 91 | 0.152605 |
| 52 | 0.004634 | 62 | 0.010139 | 72 | 0.020609 | 82 | 0.056911 | 92 | 0.169494 |
| 53 | 0.004981 | 63 | 0.010849 | 73 | 0.02262 | 83 | 0.063279 | 93 | 0.187623 |
| 54 | 0.00537 | 64 | 0.01155 | 74 | 0.024958 | 84 | 0.070704 | 94 | 0.206647 |
| 55 | 0.005831 | 65 | 0.012216 | 75 | 0.027906 | 85 | 0.079184 | 95 | 0.22589 |
| 56 | 0.006326 | 66 | 0.012952 | 76 | 0.030925 | 86 | 0.088697 | 96 | 0.245054 |
| 57 | 0.006837 | 67 | 0.013844 | 77 | 0.03414 | 87 | 0.09924 | 97 | 0.263815 |
| 58 | 0.007399 | 68 | 0.014863 | 78 | 0.03762 | 88 | 0.11048 | 98 | 0.281828 |
| 59 | 0.008033 | 69 | 0.016028 | 79 | 0.041725 | 89 | 0.123078 | 99 | 0.298738 |
| 60 | 0.008687 | 70 | 0.017329 | 80 | 0.046324 | 90 | 0.137152 | 100 | 0.316662 |

^a^Source: Period Life Table, 2021. 2024 Trustees Report. [28]

US, United States.

Table S3. Relative risks of mortality vs the general population for the hip and vertebral fracture^a^

| **Age (Years)** | **Hip First Year** | **Clinical Vertebral First Year** | **Wrist or Other Fracture, All Years** | **Hip 2+ Years** | **Clinical Vertebral 2+ Years** |
| --- | --- | --- | --- | --- | --- |
| 50 | 9.79 | 12.07 | 1.22 | 3.62 | 7.94 |
| 51 | 9.70 | 12.99 | 1.22 | 3.62 | 8.54 |
| 52 | 9.34 | 14.58 | 1.22 | 3.51 | 9.58 |
| 53 | 9.51 | 13.65 | 1.22 | 3.61 | 8.97 |
| 54 | 8.90 | 10.90 | 1.22 | 3.41 | 7.17 |
| 55 | 8.64 | 10.15 | 1.22 | 3.34 | 6.67 |
| 56 | 8.28 | 10.92 | 1.22 | 3.23 | 7.18 |
| 57 | 8.01 | 11.08 | 1.22 | 3.16 | 7.29 |
| 58 | 7.83 | 10.76 | 1.22 | 3.11 | 7.07 |
| 59 | 7.90 | 10.62 | 1.22 | 3.17 | 6.98 |
| 60 | 7.69 | 9.04 | 1.22 | 3.11 | 5.94 |
| 61 | 7.39 | 10.00 | 1.22 | 3.02 | 6.57 |
| 62 | 6.80 | 8.18 | 1.22 | 2.80 | 5.38 |
| 63 | 6.85 | 8.26 | 1.22 | 2.85 | 5.43 |
| 64 | 6.63 | 7.44 | 1.22 | 2.78 | 4.89 |
| 65 | 6.39 | 7.43 | 1.22 | 2.70 | 4.88 |
| 66 | 6.04 | 7.82 | 1.22 | 2.58 | 5.14 |
| 67 | 6.22 | 7.01 | 1.22 | 2.68 | 4.61 |
| 68 | 6.00 | 6.51 | 1.22 | 2.60 | 4.28 |
| 69 | 5.62 | 6.27 | 1.22 | 2.46 | 4.12 |
| 70 | 5.54 | 5.98 | 1.22 | 2.44 | 3.93 |
| 71 | 5.14 | 5.49 | 1.22 | 2.29 | 3.61 |
| 72 | 4.87 | 5.03 | 1.22 | 2.18 | 3.31 |
| 73 | 4.55 | 4.93 | 1.22 | 2.06 | 3.24 |
| 74 | 4.28 | 4.33 | 1.22 | 1.95 | 2.84 |
| 75 | 4.16 | 4.39 | 1.22 | 1.91 | 2.88 |
| 76 | 3.79 | 3.97 | 1.22 | 1.76 | 2.61 |
| 77 | 3.72 | 3.77 | 1.22 | 1.73 | 2.48 |
| 78 | 3.54 | 3.39 | 1.22 | 1.66 | 2.23 |
| 79 | 3.21 | 2.98 | 1.22 | 1.52 | 1.96 |
| 80 | 2.92 | 2.75 | 1.22 | 1.39 | 1.81 |
| 81 | 2.80 | 2.60 | 1.22 | 1.34 | 1.71 |
| 82 | 2.62 | 2.44 | 1.22 | 1.27 | 1.61 |
| 83 | 2.46 | 2.20 | 1.22 | 1.20 | 1.44 |
| 84 | 2.30 | 1.96 | 1.22 | 1.13 | 1.29 |
| 85 | 2.15 | 1.98 | 1.22 | 1.06 | 1.30 |
| 86 | 2.03 | 1.72 | 1.22 | 1.01 | 1.13 |
| 87 | 1.89 | 1.61 | 1.22 | 1.00 | 1.06 |
| 88 | 1.79 | 1.55 | 1.22 | 1.00 | 1.02 |
| 89 | 1.70 | 1.41 | 1.22 | 1.00 | 1.00 |
| 90 | 1.63 | 1.36 | 1.22 | 1.00 | 1.00 |
| 91 | 1.53 | 1.27 | 1.22 | 1.00 | 1.00 |
| 92 | 1.50 | 1.23 | 1.22 | 1.00 | 1.00 |
| 93 | 1.47 | 1.18 | 1.22 | 1.00 | 1.00 |
| 94 | 1.44 | 1.14 | 1.22 | 1.00 | 1.00 |
| 95 | 1.42 | 1.06 | 1.22 | 1.00 | 1.00 |
| 96 | 1.40 | 1.03 | 1.22 | 1.00 | 1.00 |
| 97 | 1.38 | 1.00 | 1.22 | 1.00 | 1.00 |
| 98 | 1.37 | 1.00 | 1.22 | 1.00 | 1.00 |
| 99 | 1.36 | 1.00 | 1.22 | 1.00 | 1.00 |
| 100 | 1.36 | 1.00 | 1.22 | 1.00 | 1.00 |

Values are rounded off to two decimal points.

^a^Source: Jonsson et al (2011) [29]

**Fig S1** Estimation of fracture incidence

**
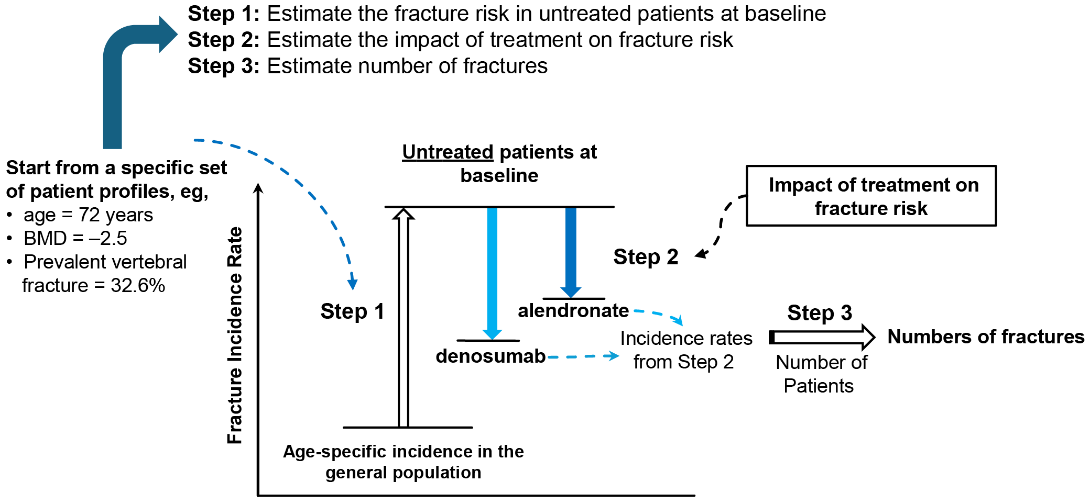
**

BMD, bone mineral density
